# Study of the Performance Enhancement of Sc-Doped AlN Super High Frequency Cross-Sectional Lamé Mode Resonators

**DOI:** 10.3390/mi14030515

**Published:** 2023-02-23

**Authors:** Meruyert Assylbekova, Michele Pirro, Xuanyi Zhao, Giuseppe Michetti, Pietro Simeoni, Matteo Rinaldi

**Affiliations:** SMART Center, Northeastern University, Boston, MA 02115, USA

**Keywords:** AlScN, MEMS resonators, Contour Lamé Mode resonators, beyond 6 GHz, piezoelectric

## Abstract

The increasing use of mobile broadband requires new acoustic filtering technologies that can operate efficiently at frequencies above 6 GHz. Previous research has shown that AlN Super High Frequency (SHF) Cross-Sectional Lamé Mode resonators (CLMRs) can address this challenge, but their performance is limited by the piezoelectric strength of AlN. In this work, we explore the use of substitutional doping of Al in AlN with Sc to enhance the $k{}_{t}{}^{2}$ values of SHF CLMRs. Our results showed that the measured $k{}_{t}{}^{2}$·$Q{}_{m}$ product of Al${}_{72}$Sc${}_{28}$N CLMRs was four times greater than that of AlN CLMRs operating at the same frequency. Additionally, the measured fractional bandwidth (FWB) of Al${}_{72}$Sc${}_{28}$N 2nd order ladder filters was 4.13%, a fourfold improvement over AlN filters with the same design. We also discuss other aspects of the technology, such as power handling, losses, and spurious mode suppression, and identify potential areas for future research.

## 1. Introduction

With almost all of the sub 6 GHz spectrum now being allocated, current bandwidth shortage has motivated the exploration of untapped frequencies beyond 6 GHz for future broadband wireless communication. A shift to higher frequency spectra is expected to deliver significant performance improvement in network capacity, data rates, and latency. Among a variety of novel 5G applications, the implementation of 5G mobile broadband imposes especially demanding specifications on Radio Frequency (RF) front end architectures. It is expected that 5G smartphones will carry over the legacy sub 6 GHz bands, which translates into an increased number of filters.

The majority of acoustic filters in the traditional RF front end architecture are formed by either surface acoustic wave (SAW) resonators or film bulk acoustic wave resonators (FBARs). These acoustic filtering technologies replaced their electromagnetic (EM) predecessors due to their small form factor, low cost and high performance. SAW filters have been utilized widely because of the simple process flow and better power handling. However, SAW filters face performance issues above 2 GHz, due to acoustic wave propagation losses, that do not affect FBAR filters until higher frequencies [1]. FBARs, on the other hand, relying on a thickness–extensional mode, provide only a single frequency per AlN deposition. Implementation of multi-frequency FBARs requires additional fabrication steps, such as mass-loading or trimming, which increase fabrication costs. Furthermore, scaling of FBARs to operate above 6 GHz incurs performance degradation, due to the reduced crystal quality in thin piezoelectric films [2]. At this point, neither technology appears to be a viable option for the implementation of future RF front ends operating above 6 GHz.

The development of acoustic filters by exploiting advancements in thin film processing and vibrational modes at higher frequencies is a promising solution to the realization of high-quality beyond-6 GHz miniature acoustic filters. For example, Super High Frequency (SHF) AlN Contour–Mode resonators (CMRs) were shown to operate in the 5–10 GHz range [3] with a loaded quality factor (*Q*) equal to 740. CMRs are attractive for operation at high frequencies given their demonstrated moderate Q values and lithographic tunability. However, their electromechanical coupling coefficient, $k{}_{t}{}^{2}$, values are limited, due to the weak piezoelectric coupling of the contour-extensional mode in AlN. Resonance at 33 GHz was demonstrated in AlN FBAR operating in a second overtone with measured loaded Q equal to 110 and 1.7% $k{}_{t}{}^{2}$ [4]. While the overtone FBAR was fabricated using thicker piezoelectric film compared to the first mode scaled to the same frequency, its $k{}_{t}{}^{2}$ significantly reduced as the mode number increased. AlN Cross-Sectional Lamé Mode Resonators (CLMRs) [5] were demonstrated to operate at 11 GHz with 1.3% and loaded Q equal to 615. Credited to the combined use of $d_{33}$ and $d_{31}$ piezoelectric coefficients of AlN [6], CLMRs offer both strong electromechanical coupling and wafer-level frequency diversity. Although AlN CLMRs can achieve $k{}_{t}{}^{2}$ as highly as AlN FBARs along with lithographic frequency tuning (delta−f), their maximum $k{}_{t}{}^{2}$ is still insufficient to accommodate ultra-wide bandwidths at high frequencies. Strong $k{}_{t}{}^{2}$ is especially useful for high frequencies where Q values are generally lower [7] and extra headroom in $k{}_{t}{}^{2}$ can be used to compensate for lower Q.

In the last decade, the MEMS community benefited from the discovery of ∼4× enhancement of electromechanical coupling through substitutional doping of Al in AlN with Sc [8]. Specifically, it has demonstrated high $k{}_{t}{}^{2}$ at resonance up to 6 GHz [9,10]. However, for resonators with frequency ($f_{res}$) above-6 GHz, $k{}_{t}{}^{2}$ values have still been below 5%, with degraded *Q*-values [11,12]. In this work, we show that by doping AlN with moderate Sc concretion (28%) and maintaining a relatively high *Q* factor, almost a 4× improvement in Figure of Merit (FoM = $k{}_{t}{}^{2}$· Q) was achieved over AlN CLMR. To pursue better designs, energy loss mechanisms relevant at frequencies beyond 6 GHz, along with power handling and spurious mode suppression, are studied. While energy loss mechanism becomes nontrivial at the SHF range, an improved resonator geometry is identified for spurious mode suppression and better power handling. The total demonstrated lithographic delta-*f* capability was over 5 GHz. Lastly, Al${}_{72}$Sc${}_{28}$N second order ladder type filter was implemented with a fractional bandwidth (FBW) exceeding that of AlN fourfold, thus opening up new possibilities for wireless communication beyond 6 GHz.

## 2. Super High Frequency AlScN CLMRs

### 2.1. $k{}_{t}{}^{2}$ and Frequency Tuning Dependence on the Design

**Figure 1:**
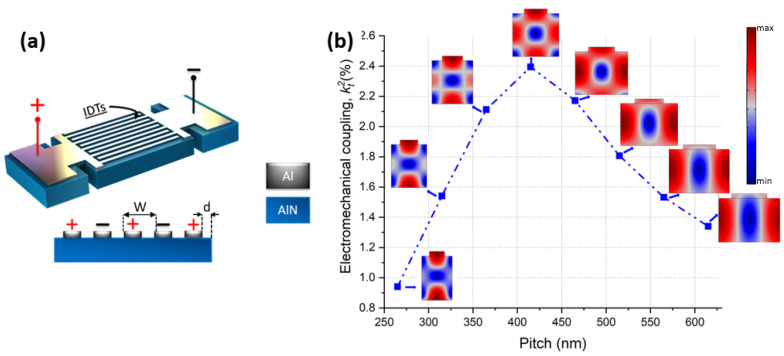
(**a**) CLMR geometry in 3D. (**b**) Simulated $k{}_{t}{}^{2}$ over a range of pitch values with corresponding mode shapes.

Cross-Sectional Lamé Mode resonators (CLMRs) introduced in [6] operate based on the piezoelectric transduction of a cross-sectional Lamé mode in an AlN plate characterized by longitudinal vibrations along both the thickness and the lateral directions. CLMRs can be excited through either thickness field excitation (TFE) requiring top and bottom interdigital metal electrodes (IDTs) or through lateral field excitation (LFE) with only top IDTs. Although a TFE scheme grants higher $k{}_{t}{}^{2}$ values, there are challenges associated with depositing good quality piezoelectric films on bottom metal electrodes [13]. An LFE scheme, on the other hand, allows for a simplified fabrication process, which reduces production costs. Figure 1a shows the 3D geometry of the LFE CLMR considered in this work. The maximum $k{}_{t}{}^{2}$ value is achieved upon excitation of the so-called non-degenerate Lamé mode. This non-degeneracy condition is met when the pitch, *W*, is approximately equal to the thickness of the piezoelectric layer, *T*. Pitch of the IDT defines the resonant frequency similarly to Lamb wave resonators (LWRs) and is equal to a half of the longitudinal wavelength (λ). However, while LWRs are based on the excitation of the lowest-order symmetric ($S_{0}$) mode solely in the lateral direction (along the x-axis), the CLMR mode is excited when $S_{0}$ mode resonance frequency matches that of $S_{0}$ mode propagating in the vertical direction (along the z-axis).

To excite the $S_{0}$ mode in both directions, $\lambda_{x}$/ $T_{AlN}$ has to satisfy the following equality as was shown in [6].

(1) $$\frac{\lambda_{x}}{T_{A}{}_{l}{}_{N}}=2\sqrt{\frac{C_{1}{}_{1}-C_{5}{}_{5}}{\left(C_{3}{}_{3}-C_{5}{}_{5}\right)}}$$

When this bidirectional resonance takes place, there is an observed peak-to-peak displacement along lateral ($\mu_{x}$) and vertical ($\mu_{z}$) directions that are approximately the same. This mutual displacement takes advantage of both, $d_{31}$ and $d_{33}$, piezoelectric coefficients resulting in higher $k{}_{t}{}^{2}$ compared to CMRs that rely solely on $d_{31}$ coefficient [6]. Figure 1b demonstrates the simulated $k{}_{t}{}^{2}$ over a range of pitch values, while AlN and Al top electrode thickness was fixed at 383 nm and 40 nm, respectively. Dimensions of the pitch values and AlN thickness were chosen so to achieve resonance at around 11 GHz. The same figure also shows the vibrational modes corresponding to each pitch value. Maximum $k{}_{t}{}^{2}$ equal to 2.38 % was achieved when *W* = 415 nm. The same figure shows an interesting change in the mode shape as the pitch value changed. When *W* > 415nm, the mode shape gradually resembled that of the contour mode. This happened because the lateral electric field became stronger compared to the electric field in the vertical direction. Conversely, when *W* < 415 nm, the lateral field strength reduced and the mode shape gradually started to resemble the thickness mode.

**Figure 2:**
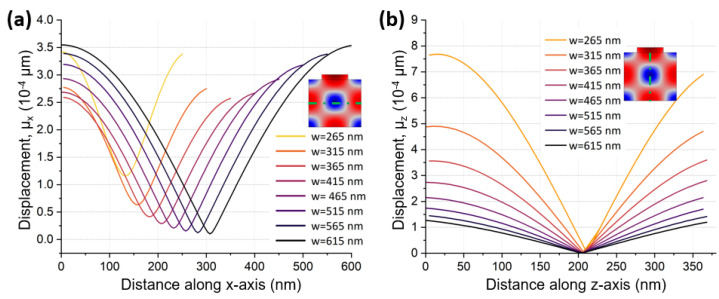
(**a**) Simulated displacement ($\mu_{x}$) along the dotted cut line on the x-axis. (**b**) Simulated displacement ($\mu_{z}$) along the dotted cut line on the z-axis. Data is simulated in Comsol®2D (ver. 5.6, Boston, MA, USA) for a fixed AlN and top metal thicknesses (383 nm and 40 nm, respectively).

The aforementioned observation could be supported through a visual comparison of displacement magnitudes in a single unit cell with fixed AlN thickness and varying pitch length. Figure 2a plots $\mu_{x}$ for each pitch value. As expected, the magnitude of $\mu_{x}$ increased with increased pitch length. Conversely, Figure 2b shows that displacement in the thickness direction, $\mu_{z}$, became larger as the pitch length became smaller. Slight deviation from the optimal $\lambda_{x}$/ $T_{AlN}$, however, can be used for lithographic frequency tuning.

**Figure 3:**
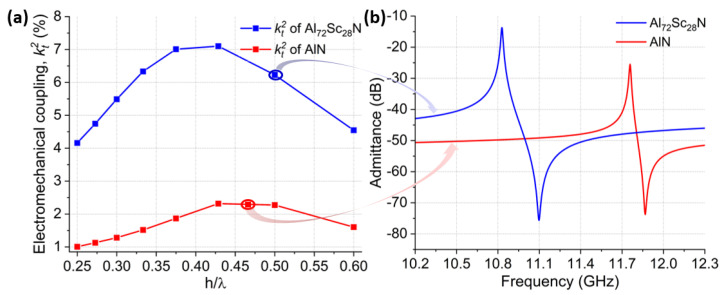
(**a**) Simulated $k{}_{t}{}^{2}$ of AlN (red) and AlScN (blue) over a range of $h/\lambda_{x}$ values. (**b**) admittance response of AlN and Al${}_{72}$Sc${}_{28}$N CLMRs operating around 11 GHz with the respective arrows indicating expected $k{}_{t}{}^{2}$ values.

Similarly, there was optimal $\lambda_{x}$/ $T_{AlScN}$ when doping AlN with Sc. Due to increased piezoelectric strength with the addition of Sc, optimal $\lambda_{x}$/ $T_{AlN}$ could give significantly higher $k{}_{t}{}^{2}$ values in comparison to AlN. Akiyama et al. demonstrated that when AlN was doped with Sc, $d_{33}$ piezoelectric coefficient of AlScN was 500% higher than $d_{33}$ of AlN for Sc concentrations between 40% and 45% [8]. The CLMRs reviewed in this study were based on AlScN with 28% Sc concentration. The predicted maximum $k{}_{t}{}^{2}$ for 28% Sc concentration was nearly three-fold that of AlN, as shown in Figure 3a, where $k{}_{t}{}^{2}$ was simulated over a range of thickness over wavelength (*h*/$\lambda_{x}$) ratios for both AlN and Al${}_{72}$Sc${}_{28}$N CLMRs. Specifically, the maximum $k{}_{t}{}^{2}$ of Al${}_{72}$Sc${}_{28}$N CLMRs was 7.1%, in comparison to 2.38% for the AlN CLMRs. This work targeted resonance frequency around 11 GHz with the expected admittance response shown in Figure 3b. Since Young’s modulus decreases with increased Sc content, the resonance frequency of AlScN devices is lower than that of AlN resonators for the same pitch dimensions.

While it is possible to accurately target a specific frequency and FoM, spurious modes appearing near the resonance are often challenging to predict and, ultimately, degrade filter performance. Here, we draw attention to a specific CLMR design parameter, namely, the “overhang”, which is a dimension measured from the edge of the outermost electrode finger to the edge of the piezoelectric plate and is labeled as *d* in Figure 1a. It has been shown that the dimension *d*, if improperly set, can contribute to the generation of unwanted spurious modes [14]. The main mode of the CLMR corresponds to a bidirectional coupling of acoustic waves traveling in lateral and vertical directions, which are in-phase. Dimension *d* needs to be such that it guarantees maximum displacement at the reflection boundary and in-phase displacement in both directions. It is demonstrated that in-phase condition was disrupted for a specific set of *d* values, resulting in the main mode being split into two smaller ones, as the trapped energy needed to be redistributed. The in-phase condition was luckily restored for a different set of *d* values.

SHF CLMRs are in the early stage of their development and need to be studied more comprehensively. Hence, along with the demonstration of good performance at super high frequency this work also encompasses a number of studies, such as a nonlinearity study and a study on relevant energy losses. Performance metrics, such as power handling and temperature coefficient of frequency, are crucial for successful commercialization and are also assessed in this work. Additionally, frequency tunability and impedance scaling are reviewed, along with a demonstration of filter responses of both SHF AlN and AlScN ladder filters.

### 2.2. Quality Factor

Large *Q* factor is indicative of low dissipation in MEMS resonators and is defined as the ratio of the stored energy over the dissipated energy per harmonic cycle [15].

(2) $$Q=2\pi \frac{E_{stored}}{E_{dissipated}}$$

Increasing the *Q* factor of MEMS resonators is essential to achieve a low insertion loss in filters [16] and low phase noise in oscillators [17]. The damping coefficient, γ, is a measure of the linear dissipation in a mechanical structure and can be expressed as

(3) $$\gamma =\frac{\sqrt{km}}{Q}$$

Equation (3) implies that a higher frequency resonator made of a soft material exhibits a smaller *Q* factor compared to its larger and stiffer counterpart. For fixed *m* and *k* coefficients, the *Q* factor can be increased by engineering the device geometry to reduce the dominant dissipative mechanisms. From Equation (2) it is apparent that, if no external energy is supplied or removed from the system, the *Q* factor is inversely proportional to the losses within the resonant structure. Several losses can affect the magnitude of the *Q* factor in a MEMS resonator and can be summarized as

(4) $$\frac{1}{Q}=\frac{1}{Q_{el}}+\frac{1}{Q_{gas}}+\frac{1}{Q_{anchor}}+\frac{1}{Q_{TED}}+\frac{1}{Q_{p-p}}$$

The losses dominating in AlScN CLMRs operating in the SHF range could be due to a single dominant dissipative mechanism or due to a combination of a few of them.

Electrical losses (1/$Q_{el}$) are straightforward in definition and imply dissipation of electrical energy into heat in the metal electrodes. In general, thicker and lower resistance metals help to minimize these losses. Proper design of the electrodes can also aid in the reduction of such losses. In particular, the resistance of a metal routing ($R_{s}$) in a fully anchored LFE CLMR is simply estimated as *R* = ρL/$Wt_{m}$, where ρ is the metal density, *L* and *W* is length and width, and $t_{m}$ is the metal thickness. The resistance of metal IDTs can be represented as a parallel combination of resistances of each metal finger with the anchor as the connecting node. The total resistance of the metal IDTs can be expressed as

(5) $$R_{IDT}=\frac{L_{e}\rho }{W_{e}t_{m}N}$$

where $L_{e}$, $W_{e}$ and N are the length, pitch and number of IDT fingers, respectively. Due to the large aspect ratio ($L_{e}/W_{e}$) of the IDTs, $R_{IDT}$ is expected to be the dominant part in the $R_{s}$ estimation.

Viscous losses (1/$Q_{gas}$) are due to the transfer of some of the resonator’s kinetic energy to the surroundings when the resonator’s surface interacts with the ambient gas molecules. This type of loss is significant for low frequency relatively large resonant structures with large separation between the vibrating body and the substrate [18]. As the surface to volume ratio of the resonant device scales down, this type of loss becomes less dominant [19].

Anchor losses (1/$Q_{anchor}$) are caused by the leak of the acoustic energy through the anchors that attach the resonator to the substrate. Anchor losses have been shown to be significant for low frequency resonators [20,21], while for higher frequency resonators, when the acoustic wavelength becomes much smaller than the resonator size (>1 GHz), they become insignificant [21].

Thermoelastic damping (1/$Q_{TED}$) is a type of loss that happens through an irreversible heat flow from the local temperature gradient generated because of the induced volume change when the elastic solid vibrates. For example, compression of a solid generates increase in the temperature, while expansion results in the decrease of the local temperature. Conveniently, in nondegenerate Lamé modes, while there is an expansion in one direction of motion, there is an equal contraction in the orthogonal direction. This leads to an interesting feature of the Lamé mode in having practically zero TED because of the zero net volume change [22]. The same might not hold true for the degenerate Lamé mode, which has an unequal displacement in orthogonal directions. TED, however, accounts for only 4% of the losses in semiconductor and dielectric devices vibrating in the extensional mode, while in metals about 50% of the losses was found to be due to the TED [23]. Segovia-Fernandez et al. [24] demonstrated TED dependence on thermal characteristics and the geometry of the metal electrodes (metal coverage) of AlN CMRs and its reduction at lower operating temperatures.

Phonon−phonon interaction-induced loss (1/$Q_{p-p}$) is another thermo-mechanical loss that has reciprocal dependence on the temperature, as in the case of TED. The two, however, differ in the way the equilibrium state of phonons is attained after being subjected to strain. In the TED case, equilibrium is achieved through the diffusive transport of heat, as opposed to the ballistic transport of phonons between hot and cold regions for loss due to phonon–phonon interactions [25]. Thus, 1/$Q_{p-p}$ loss is considered local in its nature and is independent of the device geometry [26]. Phonon–phonon interaction is, in turn, divided into two regimes, based on the magnitude of the acoustic wavelength (λ) relative to the mean free path of phonons (τ). In the so-called Akheiser regime, when λ is significantly larger than the τ, it is assumed that the acoustic waves interact with a group of phonons and change their frequencies locally. In this regime, f·Q product, which is another important figure of merit in MEMS resonators, is constant for longitudinal acoustic waves [25]. When λ becomes smaller than τ, elastic phonons (acoustic quanta) start to interact with individual thermal phonons. This regime was named the Landau–Rumer regime and is characterized by the linear dependence of f·Q product on the operating frequency [27]. The transition between the two aforementioned regimes occurs when λ = τ and is reached by either reducing λ, through scaling down of the device dimensions (e.g., pitch), or by operating the device at cryogenic temperatures. The former is possible to implement with electron-beam (e-beam) lithography while the latter is more practical, using the fact that the τ increases with decreasing temperature [28]. In this work, we used a combination of both methods, a high frequency device (small λ) operating at cryogenic temperature (increased τ) to cross the Akhiezer regime.

### 2.3. Nonlinearity and Power Handling

Maximum power handling is an important criterion for filtering applications. Nonlinearity arises when input power exceeds the resonator’s power capacity. Nonlinear response compromises signal integrity and leads to the generation of intermodulation products. Operation at high frequency, unfortunately, incurs reduced energy storage capability as the resonator size is scaled down compared to larger quartz resonators [29]. Among various sources of nonlinearity in MEMS resonators, thermal nonliterary has been identified to be the main limiter of the power handling of AlN Lamb wave resonators [30]. Thermal nonlinearity is manifested by the self-heating of the device when some of the input energy is converted to heat within the resonator body (Joule heating). Self-heating reduces the Young’s modulus of the piezoelectric film, thus lowering the resonance frequency. Downward shift in the resonance frequency, in turn, increases device impedance at a given frequency, which increases the amplitude of oscillations and results in further decrease of the resonance frequency. Non-linear response is usually captured through the amplitude versus frequency (A-*f*) response and can be described through the equation of motion of a forced Duffing oscillator:

(6) $$m\ddot{x}+\gamma \dot{x}+kx+\alpha x^{3}=F\left(t\right)$$

where *m* is the total mass, γ controls the amount of damping, *k* controls the linear stiffness and α controls the amount of non-linearity in the restoring force. When the oscillator trajectory depends on the initial conditions, the system can be assumed to be weakly non-linear and analysis can be restricted up to second-order non-linear terms. In such a case, at high enough power levels there is an observed bifurcation response in the amplitude-frequency response with two possible solutions for the equilibrium temperature near resonance. As the input power is increased further, the nonlinear cubic coefficient becomes non negligible, resulting in a disturbed resonance response with the eventual appearance of hysteresis with three solutions for the equilibrium temperature. The third order nonlinearity coefficient, α, was derived for a CMR with a bottom floating electrode in [31] and expressed as:

(7) $$\alpha =TCFR_{TH}\left(R_{M}+R_{S}\right)\omega_{0}^{2}$$

To apply this formula for LFE CLMR, Equation (7) was re-evaluated. Specifically, $\omega_{0}^{2}$ term was substituted with the linear frequency adopted from [32] for degenerate CLMR modes

(8) $$f_{r}\approx \frac{1}{2T_{AlN}}\sqrt{1+{\left(\frac{2T_{AlN}}{\lambda_{x}}\right)}^{2}}\sqrt{\frac{2C_{lat}}{4\rho }}$$

while $R_{M}$ + $R_{S}$ expression was almost unaltered from the one in [31] with the exception of substituting the $d_{3^{1}}^{2}$ coefficient with $\sqrt{d_{3^{3}}^{2}+d_{3^{1}}^{2}}$ as the CLMR mode was two-dimensional

(9) $$R_{M}+R_{S}\approx \frac{1}{n}\frac{\pi }{8}\frac{\sum T_{R,i}}{L}\frac{{\rho^{1/2}}_{eq}}{{E^{3/2}}_{eq}\sqrt{d_{3^{1}}^{2}+d_{3^{3}}^{2}}Q}$$

where *n* denotes the number of metal electrode pairs and $T_{Ri}$, $\rho_{eq}$ and $E_{eq}$ are the equivalent layer stack thickness, density and Young’s modulus, respectively. The thermal resistance, $R_{TH}$, of the narrowly anchored resonator in [31] is expressed as

(10) $$R_{TH}=\frac{L_{t}}{\sum k_{i}T_{R,i}W_{t}}$$

where $L_{t}$, $W_{t}$ and $T_{R,i}$ are the length, width and total thickness of the layers comprising anchors and $\sum k_{i}$ is the material stack conductivity. After substituting Equations (8)–(10) into Equation (7), the α coefficient can be expressed in terms of the geometry and material properties of AlN CLMRs. To simplify the analysis, $\sum T_{R,i}$ and $\sum \rho_{eq}$ were set to be equal to $T_{AlN}$ and ρ, assuming the top electrode thickness was much smaller compared to the thickness of AlN. After some simplifications, α can be expressed as

(11) $$\alpha =TCF\cdot \frac{\pi^{3}}{8n}\frac{L_{t}}{\sum k_{i}W_{t}L}\cdot \frac{1}{{E^{3/2}}_{eq}\sqrt{d_{3^{1}}^{2}+d_{3^{3}}^{2}}Q}\cdot \frac{C_{lat}}{2\rho^{1/2}}\cdot \left(\frac{1}{\sum T_{R,i}^{2}}+\frac{2}{W_{e}^{2}}\right)$$

From Equation (11) it is evident that scaling the device geometry to operate at higher frequencies ( small $T_{Ri}$ and $W_{e}$) results in increased nonlinearity. A larger device area (large *n* and *L*), and a wider and shorter anchor geometry, can be used to reduce the α coefficient. Higher *Q*, $d_{31}$ and $d_{33}$ coefficients can also aid in the reduction of nonlinearity. While often geometrical dimensions of the active area cannot be easily modified, anchor geometry is not critical and is more available for alterations. Hence, in this work we demonstrated the experimental results on the power handling of CLMRs with three different anchor widths.

## 3. Fabrication

**Figure 4:**
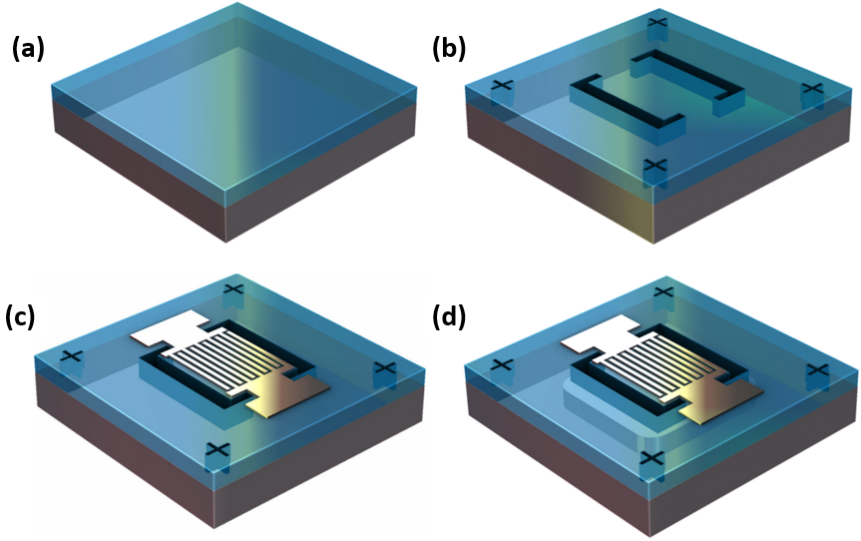
Fabrication steps of CLMR. (**a**) deposition of piezoelectric layer. (**b**) Reactive ion etching (RIE) of AlN/AlScN trenches and alignment marks. (**c**) Lift-off of 40 nm Al patterned using Electron Beam Lithography (EBL) tool. (**d**) dry release in *XeF*_2_.

The AlN and AlScN CLMRs were fabricated on a high resistivity wafer with an identical fabrication flow, as outlined in Figure 4.

**Figure 5:**
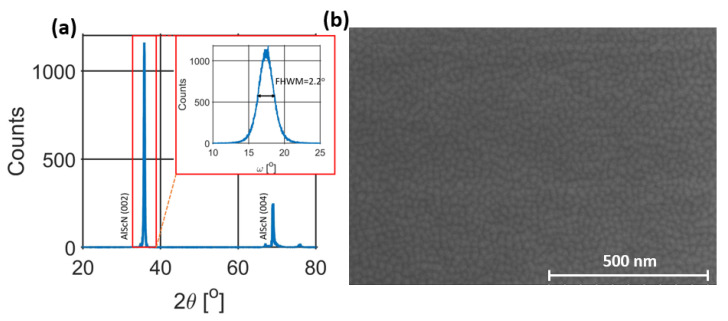
(**a**) XRD 2Θ scan patterns with Rocking curves of 002 Al_72_Sc_28_N diffraction peaks shown in the inset. (**b**) SEM micrograph of Al_72_Sc_28_N film.

An amount of 383 nm of AlN were deposited externally using the Tegal AlN Sputtering System at Carnegie Mellon University with a Full-Width-at Half-Maximum (FWHM) value of 1.5${}^{\circ }$, exhibiting excellent crystallinity. Then, 300 nm of Al${}_{72}$Sc${}_{28}$N film was deposited using an in-house EVATEC CLUSTERLINE 200 MSQ multi-source system with Al and Sc 4-inch targets. Deposition started after a long conditioning of the chamber to reach a base pressure in the 10${}^{-8}$ mbar range and after the deposition of two dummy wafers with the same process parameters as for the production wafers. In between each run, a paste–poison procedure was used to have a consistent surface on the sputtering targets. The deposition, at 350 ${}^{\circ }$C, used 900 DC W + 100 RF W on the Al target and 450 W on the Sc target. The power on the Sc target was pulsed at 150 kHz with 88% duty cycle. Nitrogen, in the amount of 20 sccm and no argon were used in the sputtering, with the substrate positioned at a distance of 33 cm from the target. Crystalline quality of the resultant film was assessed via XRD measurements, as shown in Figure 5a, where a FWHM of 2.2${}^{\circ }$ was extracted from the omega scan. Along with the good crystallinity, a Scanning Electron Micrograph (SEM) of the film revealed low to no occurrence of Abnormally Oriented Grains (AOGs), as shown in Figure 5b.

**Figure 6:**
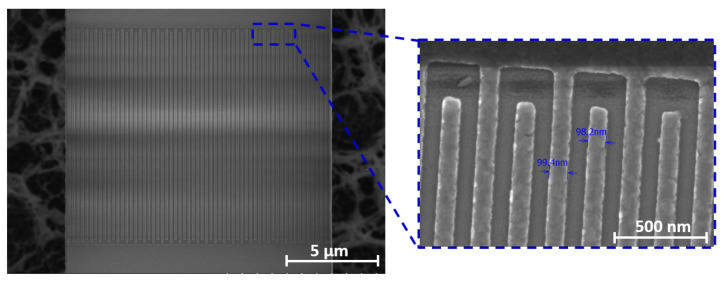
SEM micrograph of released 60-finger AlScN CLMR with the zoomed in view of ∼100 nm wide finger electrodes shown in the inset.

The piezoelectric film deposition shown in Figure 4a was followed by Plasma-enhanced Chemical Vapor Deposition (PECVD) of 700 μm of SiO${}_{2}$, used as a hard mask. The mask was then used for the etching of Al${}_{72}$Sc${}_{28}$N trenches and alignment marks (Figure 4b) used for the subsequent layer. The dry etch recipe of Al${}_{72}$Sc${}_{28}$N was similar to the one described in [10], with the exception of using 400 W bias power to get a higher sidewall angle. The remaining hard mask layer was removed in Buffered Oxide Etchant (BOE). A dry etch was performed prior to Al top electrode deposition in order to avoid metal lift-off in BOE. Next, a thin layer of PMMA was spun on the surface, followed by the top electrode pattern being transferred through e-beam exposure. Exposed regions were removed in cooled solvent to improve the resist contrast. The exposure dose and the development time were tightly controlled in order to resolve the 100 nm-wide features. shown in Figure 6, that were prone to breakage due to the high aspect ratio of the fingers. E-beam lithography was followed by thermal evaporation of 40 nm of the top Al electrode and its subsequent lift-off (Figure 4c). Finally, the devices were released in *XeF*_2_ (Figure 4d).

## 4. Results and Discussion

Resonator performance was assessed by recording the S11 parameters and converting them into the admittance response. Measurements were performed using a Vector Network Analyzer (VNA, model: Keysight PNA N5221A) with −20 dBm power level and 50 Ω port impedance. Resonator behavior was represented by an equivalent modified Butterworth–Van Dyke (mBVD) model [33], where motional ($R_{m}$, $L_{m}$, $C_{m}$) and static ($C_{0}$, $R_{o}$, $R_{s}$) lumped parameters were fitted to a given resonator S11 response. Extracting the $R_{s}$ value from the mBVD fitting required a long frequency span and was often not accurate. Instead, in this work, $R_{s}$ values were calculated based on the electrode geometry and measured Al sheet resistance, according to Equation (5), and manually fixed in the mBVD model. Excluding $R_{s}$ loading, the motional *Q* factor, $Q_{m}$, was estimated according to the following equation

(12) $$Q_{m}=\frac{2\pi f_{s}L_{m}}{R_{m}}$$

while $k{}_{t}{}^{2}$ is calculated as

(13) $$k_{t}^{2}=\frac{\pi^{2}}{8}\frac{C_{m}}{C_{0}}$$

**Figure 7:**
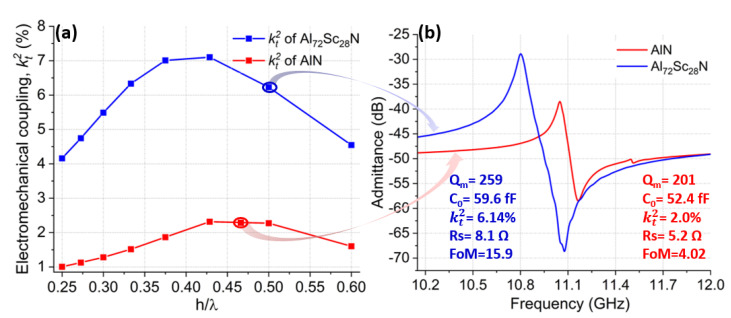
(**a**) Simulated $k{}_{t}{}^{2}$ of AlN (red) and AlScN (blue) over a range of $h/\lambda_{x}$ values. (**b**) measured admittance response of AlN and Al${}_{72}$Sc${}_{28}$N CLMRs operating around 11 GHz with respective arrows indicating expected $k{}_{t}{}^{2}$ values.

Figure 7 compares the performances of AlN and Al${}_{72}$Sc${}_{28}$N CLMRs with color-coded arrows pointing to the corresponding simulated $k{}_{t}{}^{2}$ values. To achieve similar resonant frequency AlN and Al${}_{72}$Sc${}_{28}$N film thicknesses were set to 383 nm and 300 nm, respectively, while the corresponding pitch values were 415 nm and 300 nm. Both devices were fabricated using an identical fabrication flow. Al${}_{72}$Sc${}_{28}$N CLMRs showed ∼3x higher $k{}_{t}{}^{2}$ compared to AlN CLMRs, while their $Q_{m}$ values were on par. Measured $k{}_{t}{}^{2}$ of Al${}_{72}$Sc${}_{28}$N CLMR was 6.14% while for AlN CLMR it was 2%. $k{}_{t}{}^{2}$ values for both resonators were close to what was predicted by COMSOL®2D, implying excellent film quality of both materials. $R_{s}$ value of AlN CLMR was slightly higher, due to the smaller pitch. Overall, Al${}_{72}$Sc${}_{28}$N CLMRs showed an enhanced response with an FoM of 15.9 compared to 4.02 for the AlN CLMR. ∼4x improvement in the FoM of Al${}_{72}$Sc${}_{28}$N CLMR was encouraging for the early steps of the device development.

**Figure 8:**
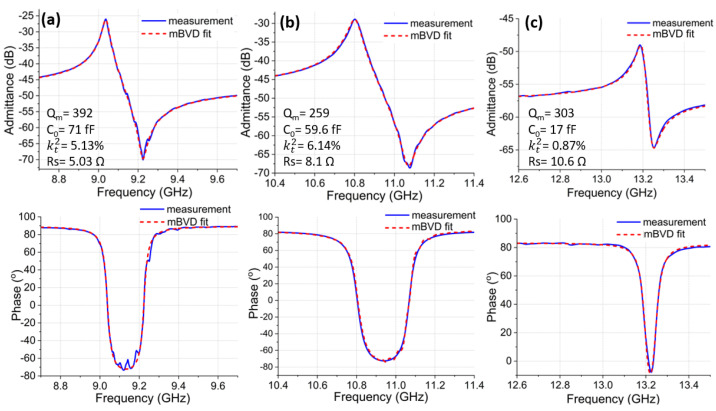
Admittance (**top**) and phase (**bottom**) response of Al${}_{72}$Sc${}_{28}$N CLMRs fitted to the mBVD model: (**a**) CLMR operating at 9.04 GHz with W${}_{e}$ = 445 nm. (**b**) CLMR operating at 10.8 GHz W${}_{e}$ = 300 nm. (**b**) CLMR operating at 13.19 GHz W${}_{e}$ = 150 nm.

Frequency tunability of Al${}_{72}$Sc${}_{28}$N CLMR was demonstrated in Figure 8, where magnitude (top row) and phase of the admittance responses measured from three CLMR devices with varied pitch were fitted to the MBVD model (dashed line). Figure 8a,b demonstrates the frequency tuning effect when h/λ was around the optimum value with $k{}_{t}{}^{2}$> 5%, while ▵f was ∼2 GHz. Figure 8c shows the potential of tuning the frequencies up to 13 GHz, which is the highest among all demonstrated piezoelectric CLMRs up to date. To note, in Figure 8c, even the $k{}_{t}{}^{2}$ degraded to ∼0.9% for the CLMR with h/λ away from the optimum, as expected, but the mechanical Q remained above 300. A quick summary of the three devices is provided in Table 1.

**Figure 9:**
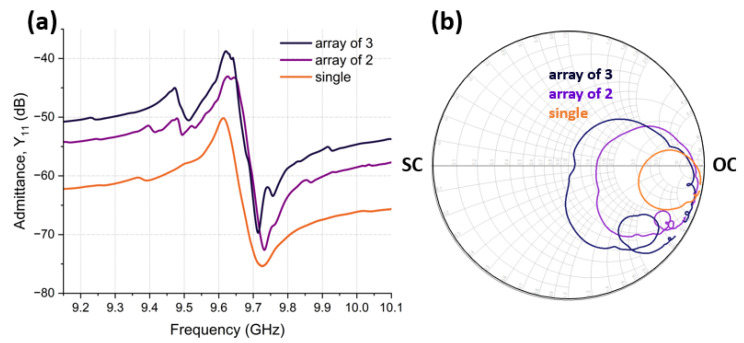
Impedance scaling: (**a**) Admittance response of a single CLMR, array of 2 CLMRs, and array of 3 CLMRs. (**b**) Impedance scaling shown on the Smith Chart.

To match to 50 Ω, around which most RF systems are designed, C${}_{0}$ can be tuned by forming arrays of identical CLMRs in parallel, and, thus, lowering the total impedance. Figure 9 demonstrates the admittance response of a single CLMR, an array of 2 CLMRs and an array of 3 CLMRs. As can be observed, C${}_{0}$ increased as the number of array elements grew, and, thus, reached the center of the Smith Chart. Specifically, the capacitance increased from 30 fF for the single device to 90 fF for the array of 3 CLMRs, corresponding to an impedance decrease from 551 Ω to 184 Ω. For ease of visualization, impedance scaling was also demonstrated on the Smith Chart (Figure 9b), where, with each additional array element, circles approached the center of the Smith Chart.

**Figure 10:**
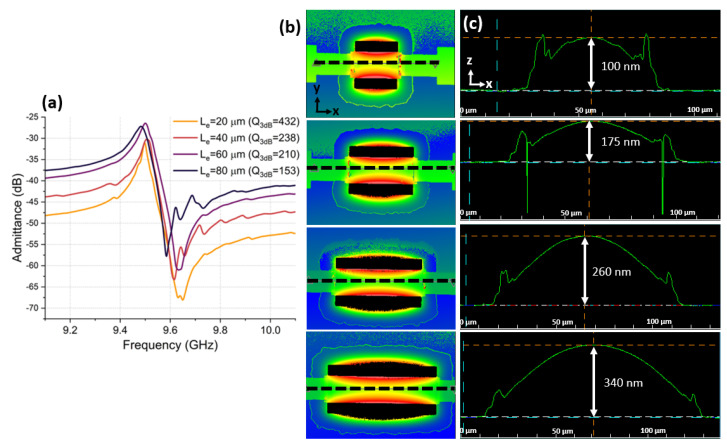
(**a**)Measured $Q_{3dB}$ of CLMRs with different $L_{e}$. (**b**) 3D ZYGO measurement of top surface view of CLMRs with different $L_{e}$. (**c**) 2D ZYGO measurement of out-of-plane bending amplitude for each $L_{e}$.

A second technique to increase $C_{0}$ involves increasing the length of the IDT fingers, $L_{e}$, keeping in mind that R${}_{IDT}$, and, thus R${}_{s}$ increases, accordingly. This impacts the 3 dB *Q* factor, $Q_{3dB}$, thereby increasing electrical losses (1/$Q_{el}$). This was confirmed in Figure 10a, where, by incrementally increasing $L_{e}$$Q_{3dB}$ decreased. Another complication associated with longer $L_{e}$ is increased susceptability to device buckling, which often results in mechanical failure of the device upon release. Stress gradients are usually the main source of bending of microbridge structures after the underlying Si is removed [34,35,36]. Out-of-plane bending increases in amplitude in released CLMRs with longer $L_{e}$, as shown in Figure 10. A 3D view of each device (Figure 10b) and out-of-plane bending amplitude (Figure 10c) were recorded using a Zygo optical profiler. Increased bending could possibly degrade *Q* factor through increased interfacial losses. However, more rigorous investigations are required to draw any conclusions.

**Figure 11:**
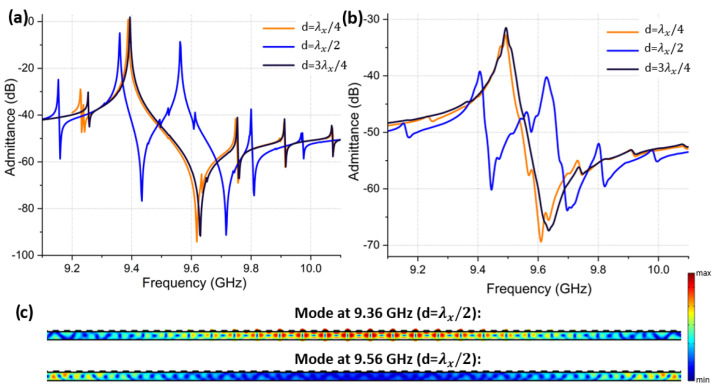
(**a**)Simulated admittance response of CLMR with *d* set to $\lambda_{x}$/4, $\lambda_{x}$/2 and 3$\lambda_{x}$/4. (**b**) Measured admittance response of CLMR with *d* set to $\lambda_{x}$/4, $\lambda_{x}$/2 and 3$\lambda_{x}$/4. (**c**) Mode shape of the two modes that appeared in the admittance response when $\lambda_{x}$/2.

As mentioned previously, another geometrical dimension of the CLMR that needs attention is the so-called “overhang” *d*. Figure 11 demonstrates simulated (a) and measured (b) admittance responses of three resonators with different *d* dimensions, which were otherwise identical. Most notably, when *d* was equal to $\lambda_{x}$/4 or 3$\lambda_{x}$/4, the admittance response was relatively clean. However, when *d* = $\lambda_{x}$/2, the main mode split into two separate ones with a similar energy distribution. Figure 11c shows the shape of the two modes that appeared in *d* = $\lambda_{x}$/2 case. A lower frequency mode appearing at 9.36 GHz still resembled the main mode because its frequency was set by both the AlScN layer and the top Al electrode. The second mode appearing at 9.56 GHz, on the other hand, originated from the sustenance of the resonance set by the non-metalized portions of the plate. Specifically, with *d* set to $\lambda_{x}$/2, cumulative non-metalized lateral distance on both ends of the plate became equal to one wavelength, thus allowing the plate edges that were free of metal to generate a higher frequency. To avoid the overlap of both frequencies that differed by the impact of metal loading, the distance *d* should be anything but an integer multiple of $\lambda_{x}$/2.

**Figure 12:**
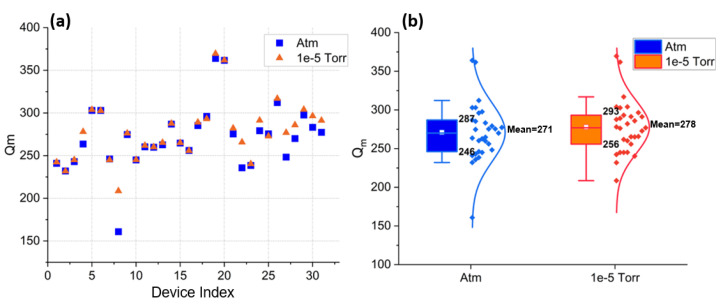
(**a**) $Q_{m}$ of 30 devices measured in air (atm) vs high vacuum (1 × 10${}^{-5}$). (**b**) The statistical summary of the $Q_{m}$ in air and in a vacuum is represented as boxes with length determined by 25% and 75% percentile and corresponding normal curve distribution.

To further investigate the sources of energy loss relevant to SHF CLMRs, 30 resonators identical to the device #1, from Table 1, were tested in air and vacuum (1 × 10${}^{-5}$ Torr), and their $Q_{m}$ values compared in Figure 12. No significant difference between the two data sets was observed. This was expected, as gas (viscous) losses become negligible as operational frequencies are increased. This was confirmed by the statistical summary shown in Figure 12b, where the mean of $Q_{m}$ in air vs vacuum was 271 vs. 278, respectively. This experiment indicated that the complexity and cost of vacuum packaging can be avoided for this device topology.

**Figure 13:**
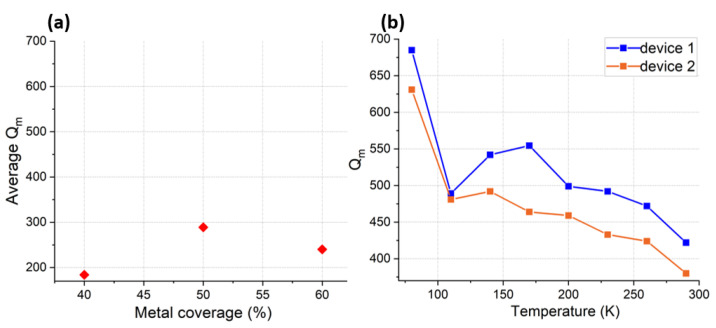
(**a**) $Q_{m}$ of CLMRs with different metal coverage ratios averaged over three identical devices. (**b**) $Q_{m}$ of two identical CLMRs measured from room temperature down to 80 K in steps of 30 K.

In the next experiment, $Q_{m}$ of CLMRs, with three different top electrode coverage ratios (40%, 50% and 60%), was extracted and averaged over 3 identical devices, as shown in Figure 13a. $Q_{m}$ did not show the trend that would prove existence of significant TED losses in metal electrodes. This was expected, since metal electrodes were placed atop minimum displacement point of the Lamé mode. To get further insight into the intrinsic material losses, two identical resonators were tested at cryogenic temperatures. Cryogenic experiments were conducted using a Lakeshore probe station with a constant chamber pressure (1 × 10${}^{-3}$ Torr). As demonstrated in Figure 13b, the $Q_{m}$ of both devices followed a similar trend as the temperature was reduced. Most interestingly, a dip in $Q_{m}$ was observed at 110 K, which coincided with the predicted Akheiser regime point for AlN [25], assuming f·Q over frequency plot of Al${}_{72}$Sc${}_{28}$N would not differ significantly from that of AlN. Deriving the exact relationship of f·Q over frequency for various Sc doping levels is under investigation. As the temperature was further decreased, $Q_{m}$ started to increase at a greater rate, signaling a transition into the Landau–Rumer regime. Overall, $Q_{m}$ showed an almost 2× improvement in magnitude compared to the $Q_{m}$ measured at room temperature. However, a weak dependence of $Q_{m}$ on temperature until 110 K implied the existence of other loss mechanisms, which shall be a subject of future research.

**Figure 14:**
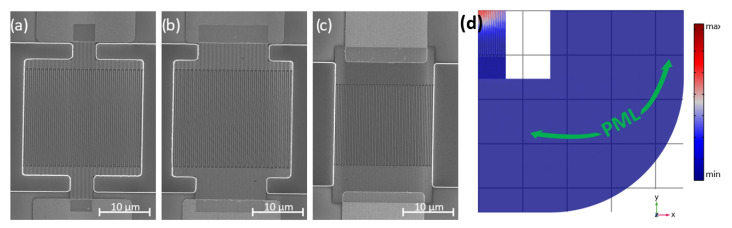
SEM micrographs and admittance responses of the three devices with different anchors: (**a**) SEM of CLMR with narrow anchor. (**b**) SEM of CLMR with medium anchors.(**c**) SEM of CLMR with wide anchors. (**d**) Total displacement amplitude at resonance frequency of fully anchored CLMR simulated in COMSOL®3D.

**Figure 15:**
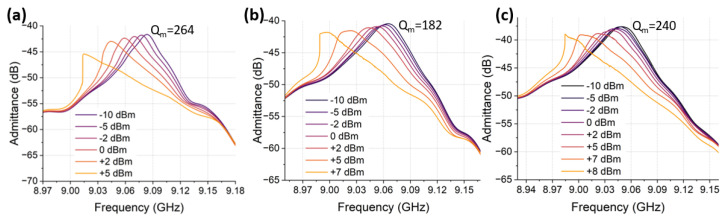
A-*f* response of SHF CLMRs at different power levels (−10 dB to +8 dB): (**a**) A-*f* response of CLMR with narrow anchors. (**b**) A-*f* response of CLMR with medium anchors. (**c**) A-*f* response of CLMR with wide anchors.

Next, we examined the impact of the anchor design on the mechanical anchor loss and power handling of SHF CLMRs. The three resonators that were tested are depicted in Figure 14a–c. The resonator with wide anchors, shown in Figure 14c, was identical to the device#1 from Table 1, while the other two also had similar dimensions, with the exception of the anchor widths. As mentioned previously, anchor losses are not considered to be significant at high frequencies (>1 GHz), which was confirmed by looking at the total displacement amplitude of the fully anchored CLMR simulated in COMSOL®3D, shown in Figure 14d, with a perfectly matched layer (PML) used to create absorbent boundary conditions. Given the available computer specifications, only a quarter of a 30-finger CLMR could be simulated with a finely resolved mesh (minimum element quality >0.1). As can be noticed, the displacement was localized around the center of the resonator’s active area with zero displacement amplitude found at the anchors. Such a feature rendered minimum anchor loss in the mechanical domain through the anchors, which was indicated as the legends, shown in Figure 15. The $Q_{m}$-value did not show a significant variation among the three of these designs. This promises that there is a good opportunity to have large anchors for better power handling without sacrifice of the quality factor.

Given the above, we could continue to evaluate the nonlinearity and power handling among these three different anchor designs by examining the response of the duffing-like phenomenon at increased power levels. Figure 15 shows a zoomed-in view of the A-*f* response evolution as the input power, P${}_{in}$, was swept from −10 dB to +5, +7, and +8 dB over 200 MHz, with 22,222 points for CLMRs with narrow, medium and wide anchors. To fulfil the steady-state condition, the IF bandwidth was set to 400 Hz to ensure the sampling time was bigger than the thermal time constant. In this way, both thermal nonlinearity and mechanical nonlinearity could be assessed [31]. The onset of the nonlinear behavior for the narrow anchor design (Figure 15a) happened when P${}_{in}$ was around 0 dBm and at +5 dBm a clear bifurcation in the A-*f* response was observed. For the medium anchor design (Figure 15b), the onset of the nonlinear behavior and the subsequent bifurcation took place when P${}_{in}$ was equal to +5 dBm and +7 dBm, respectively. Lastly, for the wide anchor design (Figure 15c) the nonlinear response became apparent between +5 dBm and +7 dBm with a clear bifurcation happening when P${}_{in}$= +8 dB. As expected, among the three anchor designs, the wider anchors provided the CLMRs with better power handling, which also confirmed earlier-derived analytical expressions for the third order nonlinearity coefficient.

**Figure 16:**
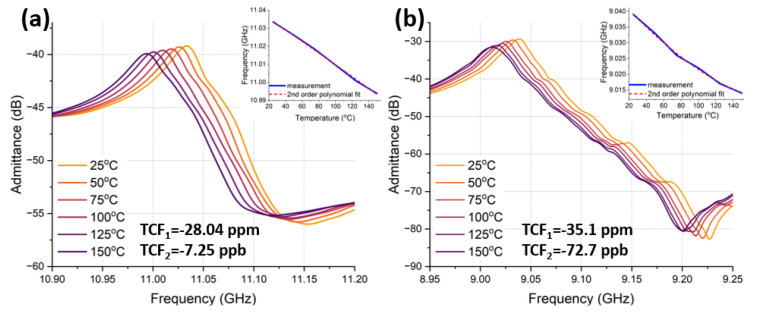
1st and 2nd order TCF measured for: (**a**) AlN CLMR. (**b**) Al${}_{72}$Sc${}_{28}$N CLMR.

Specifically, thermal nonlinearity strongly depends on the temperature coefficient of frequency (TCF), as can be seen from Equation (11). Thus, besides geometrical design, the TCF of our fabricated devices were investigated and assessed. In particular, 1st and 2nd order TCFs were compared between Al${}_{72}$Sc${}_{28}$N and AlN CLMRs. Figure 16 depicts the admittance response recorded over a 25–150 ${}^{\circ }$C temperature range for AlN (a) and Al${}_{72}$Sc${}_{28}$N (b) CLMRs. TCF was calculated as the slope of ▵f over ▵T and fitted to a 2nd order polynomial fit, shown in the insets, to extract linear and quadratic TCF coefficients that corresponded to the 1st and 2nd order TCF, respectively. The 1st order TCF of AlN was −28.05 ppm, which was on par with other AlN Lamb wave resonators [37]. Al${}_{72}$Sc${}_{28}$N 1st order TCF was −35.1 ppm and was also comparable to other AlScN resonators with similar Sc doping levels [38]. Al${}_{72}$Sc${}_{28}$N temperature stability was degraded compared to AlN, due to increased material softening with the addition of Sc. Nevertheless, thermal compensation techniques have been widely studied [39,40,41,42] and can be adopted to simultaneously improve frequency stability and power handling.

**Figure 17:**
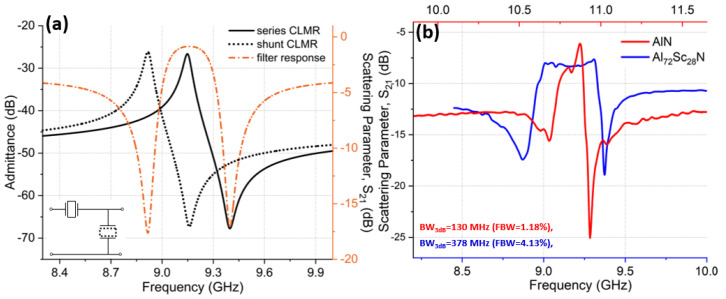
Simulated and measured filter response of AlN and Al${}_{72}$Sc${}_{28}$N filters: (**a**) Comsol 2D simulated admittance of Al${}_{72}$Sc${}_{28}$N series and shunt CLMRs along with the S21 scattering parameter of the 2nd order Ladder filter. (**b**) Measured S21 filter response of AlN and Al${}_{72}$Sc${}_{28}$N resonators.

Lastly, we demonstrated a monolithically integrated 2nd order ladder filter composed of Al${}_{72}$Sc${}_{28}$N CLMRs, along with its AlN counterpart. Figure 17a shows the simulated admittance and filter responses of the constituent resonators (black straight and dashed lines) and filter (orange line) itself. The filter response was centered at the resonance frequency of the series resonator ($f_{s}∼9.15$ GHz). To maximize transmission and bandwidth, the anti-resonance frequency of the shunt CLMR was made to overlap with $f_{s}$ by setting the pitch of the series and shunt resonator to be equal to 445 nm and 430 nm, respectively. Following these design parameters, we fabricated a pair of such filters with identical geometry combinations using AlN and AlScN. As demonstrated in Figure 17b, the measured $S_{21}$ of the Al${}_{72}$Sc${}_{28}$N filter showed a 3 dB fractional bandwidth (FBW) of ∼4.13%, which was ∼4 times larger than the AlN implementation with a FBW of ∼1.18%. Note that the AlN filter had a higher center frequency because the resonance frequencies of the AlN CLMRs were higher than the AlScN ones for the same design, due to the variation of the materials’ elastic properties. Without any matching network, respective I.L. values of Al${}_{72}$Sc${}_{28}$N and AlN filters were −7.7 dB and −6.12 dB, which can be improved through impedance matching as well as provisional improvement in Q. The measured out of band rejection (OBR) of the AlN filter was −13 dB and −12.7 dB as measured on both sides of the frequency spectrum, while the Al${}_{72}$Sc${}_{28}$N filter OBR was −12.4 dB and −10.7 dB. Modest OBR was attributed to the small capacitance ratio of the shunt and series resonators (72 fF vs. 80 fF for Al${}_{72}$Sc${}_{28}$N) as well as the low filter order. Nevertheless, remarkable enhancement in the FBW of SHF CLMR filter was demonstrated by doping AlN with moderate Sc doping level (28%), signifying greater improvement should be expected at increased Sc concentrations.

## 5. Conclusions

In this work, Al${}_{72}$Sc${}_{28}$N CLMRs were successfully scaled to operate beyond the 6 GHz frequency range with a simple three-mask fabrication process. The FoM (∼18) of Al${}_{72}$Sc${}_{28}$N CLMRs surpassed that of AlN CLMRs operating around the same frequency (∼11 GHz), by four times. In addition, a thorough analysis of loss mechanisms, power handling capacity, and frequency tuning capability were conducted to optimize our design and pave paths towards future research. Importantly, intrinsic losses were studied, and the proximity of the Akhieser regime was identified at cryogenic temperatures. Further scaling to higher frequencies encouraged entering the Landau–Rumer regime at room temperature, where f·Q increased linearly with frequency. Lastly, an Al${}_{72}$Sc${}_{28}$N 2nd order ladder filter demonstrated 4.13% FBW, which was a fourfold improvement over an identical AlN filter. The demonstrated performance enhancement achieved through the substitutional doping of Al in AlN with Sc is promising to enable advances in filtering technologies beyond 6 GHz.

## Figures and Tables

**Table 1:** Summary of the three CLMRs reported in Figure 8.

Device #	Pitch	Frequency	$k{}_{t}{}^{2}$	$Q_{m}$
1	445 nm	9.04 GHz	5.13%	392
2	300 nm	10.8 GHz	6.14%	259
3	150 nm	13.19 GHz	0.87%	303

## Data Availability

Data are available within the article.
